# Feasibility analysis of bone density evaluation with Hounsfield unit value after fibula flap reconstruction of jaw defect

**DOI:** 10.1186/s40902-023-00397-3

**Published:** 2023-08-30

**Authors:** Yihui Yang, Yifan Kang, Yifan Yang, Mengkun Ding, Xiaofeng Shan, Zhigang Cai

**Affiliations:** Department of Oral and Maxillofacial Surgery, Peking University School and Hospital of Stomatology, National Center of Stomatology, National Clinical Research Center for Oral Diseases, National Engineering Research Center of Oral Biomaterials and Digital Medical Devices, No. 22 South Avenue, Zhongguancun, Haidian District, Beijing, 100081 People’s Republic of China

**Keywords:** Mandible reconstruction, Bone density, Vascularized fibula free flap, Computed tomography, Bone resorption, Dental implantation, Jaw reconstruction, Dental rehabilitation

## Abstract

**Background:**

Implant-supported dentures have become an essential means of restoring occlusal function after jaw reconstruction. Bone mineral density (BMD) may influence the success rate of implant denture restorations. This study aimed to explore whether the Hounsfield unit (HU) value can be used to monitor the changing trend of fibular BMD after jaw reconstruction.

**Results:**

A total of 54 patients who underwent maxillar/mandibular reconstruction with a fibula flap were included in this study. There was a significant correlation between the HU value and BMD at 1 week, 3 months, and 6 months after surgery, and both were significantly correlated with follow-up time. The difference between each pair of absorption rates (DAR) was less than 10% in 66.7% and 75.9% of patients at 3 and 6 months; however, the DAR was more than 20% in 12% and 13.8% of patients at 3 and 6 months, respectively.

**Conclusions:**

There is a significant correlation between HU value and BMD. The HU value can be used to roughly reflect the fibular BMD changing trend in a group of patients as opposed to an individual, and the HU value is not equivalent to BMD.

**Trial registration:**

ChiCTR, ChiCTR2300069661, retrospectively registered on 22 March 2023. Retrospectively registered, https://www.chictr.org.cn/showproj.html?proj=188953.

## Background

Oral and maxillofacial tumors, trauma, and inflammation often cause jaw defects that seriously affect patients’ quality of life [1]. Reconstruction of the shape and function and restoration of the quality of life and social activities of patients have been difficult problems for oral and maxillofacial surgeons. Currently, the main method for the reconstruction of large-area jaw defects is the vascularized free bone flap, and the workhorse is the vascularized fibula flap [2].

Owing to the lack of a normal jaw and surrounding soft tissue in patients undergoing jaw reconstruction, restoring normal occlusal function with removable dentures is difficult. Implant-supported dentures have become an essential means of restoring occlusal function [3]. Bone resorption may occur after fibular flap transplantation, which reduces the long-term survival rate of the implant and ultimately affects the success rate of implant denture restoration [4]. Bone mineral density (BMD) is an important index for predicting long-term survival rates of implants [5]. Quantitative computed tomography (QCT) is a relatively accurate method for measuring BMD [6]. QCT has not yet been fully popularized; therefore, the Hounsfield unit (HU) value is considered a potential substitute for estimating BMD. The HU value is calculated by analyzing the linear attenuation density of different human tissues in computed tomography (CT) images using specific software [7]. It is not the actual BMD; however, compared with QCT, the HU value is easier to measure. Therefore, whether the HU value can be used to estimate the BMD of the grafted bone and observe its changing trend has gradually become a topic worthy of discussion.

## Methods

Based on the above reasons, this study aimed to measure BMD and HU values after maxillary and mandibular reconstruction with the fibula, analyze the correlation between them, and discuss whether the HU value can be used to monitor the changing trend of fibular BMD after jaw reconstruction.

This study included patients who underwent maxillar/mandibular reconstruction with a fibular flap at the Department of Oral and Maxillofacial Surgery, Peking University School of Stomatology, Beijing, China, between September 2021 and September 2022. The inclusion criteria were as follows: (1) at least one segmental fibula used to repair the alveolar ridge area and (2) spiral CT examination performed 1 week after the surgery and at least one CT examination performed 3–9 months after the surgery. The exclusion criteria were as follows: (1) patients with titanium plate exposure, infection, or flap failure after surgery; (2) patients with bone metabolism disease; (3) patients who could not undergo CT examination for some reason; and (4) patients who received postoperative radiotherapy/chemotherapy. This study adhered to the principles of the Declaration of Helsinki and was approved by the Institutional Ethics Committee of Peking University School and Hospital of Stomatology (Approval No: PKUSSIRB-202282159). Informed consent was obtained from those who participated.

### Spiral CT photography

CT images [120 kV, 25 mA, section width = 1.25 mm] were acquired immediately after the surgery (1 week after the surgery) and 3 and 6 months after the surgery. The patient was placed in a supine position, the head was placed at the center of the headrest, and the Frankfurt Plane was kept vertical to the ground. The patients were instructed to occlude the posterior teeth. A calibration phantom was used to calibrate the BMD values obtained using the QCT software each day to obtain the most accurate measurement data. The scanning data were simultaneously transmitted to the Picture Archiving and Communication System (PACS) in Digital Imaging and Communications in Medicine (DICOM) format and to the QCT workstation in QCT file format. The patients underwent spiral CT (BrightSpeed Edge Select 16 slice, Ge, USA, 120 kv, 25 mAs) of the oral and maxillofacial regions.

### HU value measurement

The fibula was observed using a bone window (800 HU), and the HU value was measured using the PACS software (Carestream Health Inc., Rochester, NY, USA).

### Measurement method

**Fig. 1:**
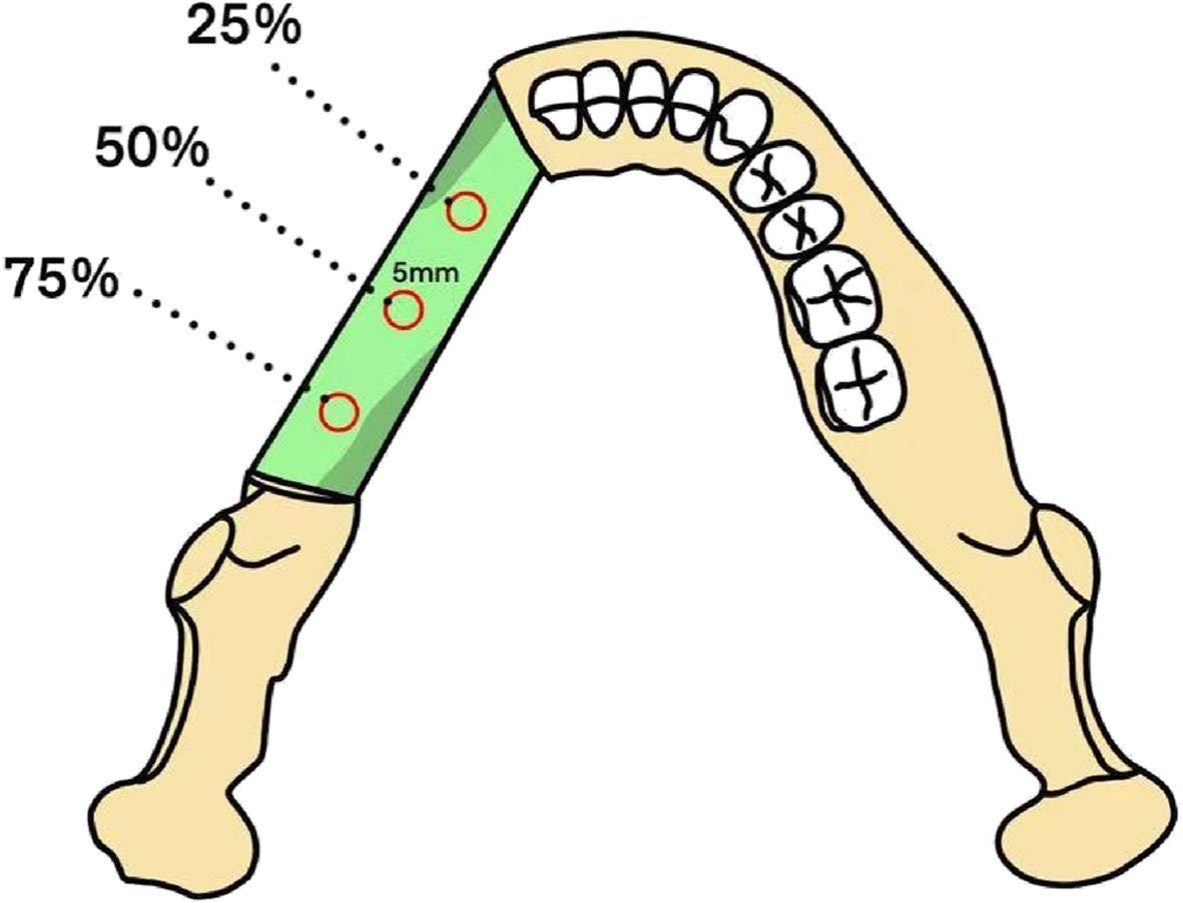
ROI of measurement. HU value and BMD were measured at 25%, 50%, and 75% of the fibula long axis, and the ROI diameter was 5 mm (5 ± 0.5 mm). ROI, region of interest; BMD, bone mineral density; HU, Hounsfield unit

The observation plane was adjusted. First, the cross-section was parallel to the occlusal plane, and the sagittal plane was perpendicular to the cross-section and parallel to the midline of the maxillary dentition. The sagittal plane was then moved to the fibula reconstruction site, and the cross-sectional and coronal plane directions were adjusted to be consistent with the long axis of the fibula. Subsequently, a circular region of interest (ROI) with a diameter of approximately 5 mm (5 ± 0.5 mm) was created on the cross-section. The center of the circle coincided with 25%, 50%, and 75% of the length of the fibular axis, and the HU value of the corresponding part was measured (Fig. 1). We measured each point three times and calculated the average value as the final outcome to minimize the errors.

### BMD value measurement

**Fig. 2:**
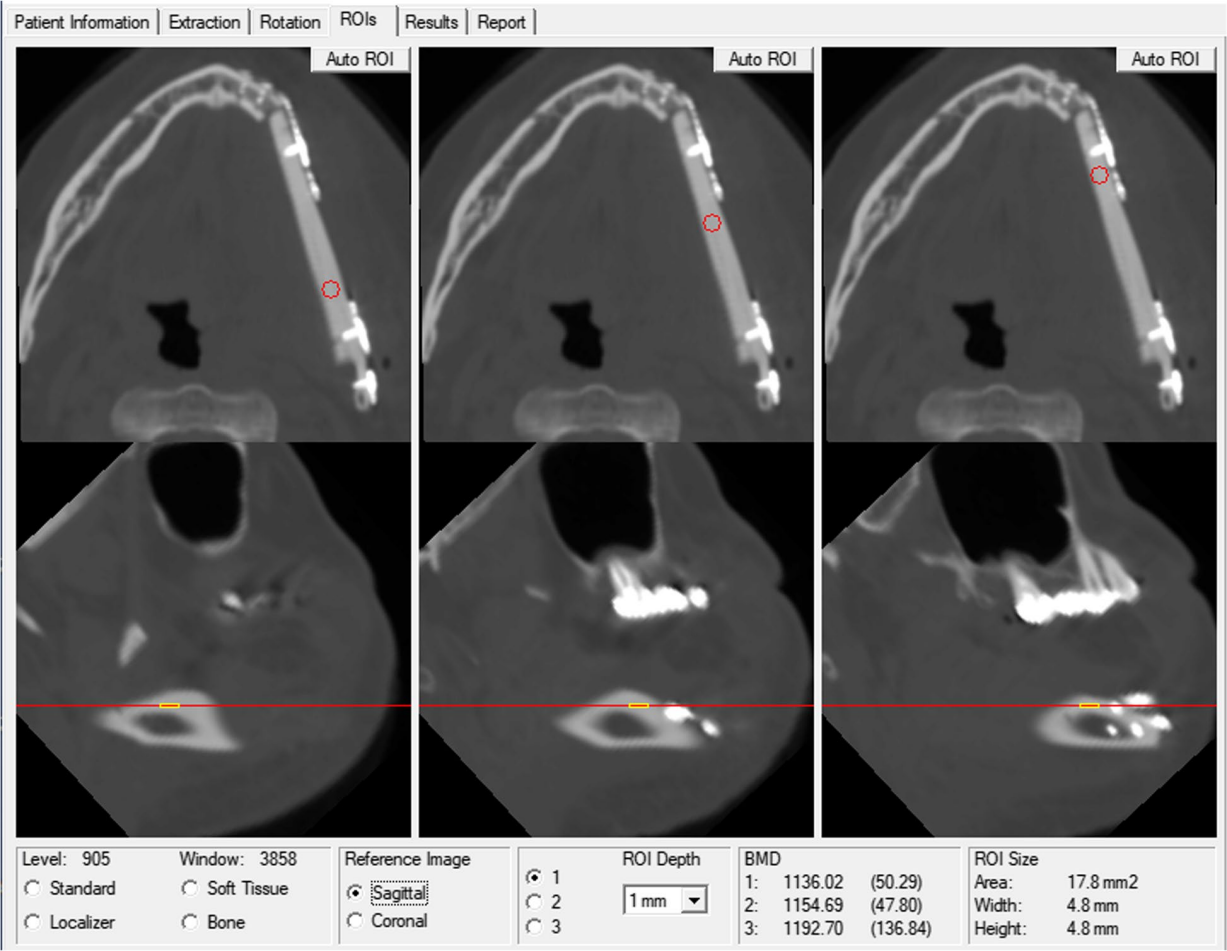
BMD measurement. The figure shows the average BMD measured at different positions 1 mm below the uppermost point of the fibular alveolar side. BMD, bone mineral density

Using the same CT image, the fibula was measured again using the QCT Pro 6.1 software (Mindways Software, Inc., Austin, TX, USA), and the corresponding BMD was obtained. The measurement method was as follows: first, the *X-*axis of the sagittal plane was adjusted to be parallel to the occlusal plane in the “rotation” interface, and then, the *Y*-axis of the cross-section was adjusted to be parallel to the midline of the dental arch of the jaw. The sagittal plane at the level of the transplanted fibula was adjusted, and the *X*-axis of the sagittal plane was adjusted parallel to the long axis of the fibula. Then, the cross-sectional level was adjusted to the uppermost point of the fibula alveolar side (superior side when the fibula was used to reconstruct the mandible and the inferior side when it was used to reconstruct the maxilla), and the 25%, 50%, and 75% sites corresponding to the axial length of the graft bone flap were used as the measurement sites. Then, a circular ROI was manually selected with a diameter of approximately 5 mm and a depth of 1 mm on the “ROIs” interface. The center of the ROI coincided with the yellow criss-cross selected on the “rotation” interface. The position in the sagittal plane was 1 mm adjacent to the uppermost point of the fibular alveolar side, and the corresponding BMD values were obtained (Figs. 1 and 2). The calculation of BMD was the same as that of the HU value. In this section, we measured each point three times and calculated the average value as the final outcome to minimize errors as much as possible.

**Fig. 3:**
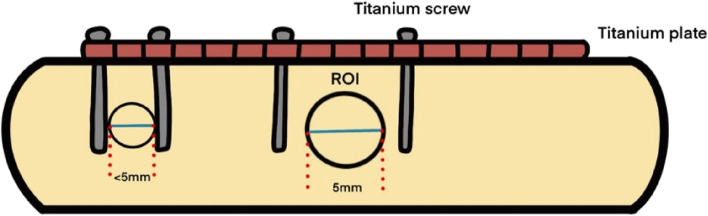
Method of adjusting and selecting ROI diameter. If we chose 5 mm as the diameter of ROI when the actual distance between two adjacent titanium nails was less than 5 mm, then the result might be influenced as ROI involved the metal artifacts. Therefore, the actual distance between two adjacent titanium nails was chosen as the final diameter of ROI in such circumstances. However, if the distance between adjacent titanium nails was more than 5 mm, then 5 mm was selected as the diameter of ROI routinely. ROI, region of interest

In the process of selecting the ROI, the effects of the titanium plate, titanium nail, or implant on the measurement may be encountered. For example, sometimes the measurement sites were located between two adjacent titanium nails. If we chose 5 mm as the diameter of ROI when the actual distance between two adjacent titanium nails was less than 5 mm, then the result might be influenced as ROI involved the metal artifacts. Thus, the diameter of ROI should be adjusted in case such an error occurred. Therefore, the actual distance between two adjacent titanium nails was chosen as the final diameter of ROI in such circumstances. However, if the distance between adjacent titanium nails was more than 5 mm, then 5 mm was selected as the diameter of ROI routinely (Fig. 3).

### Index calculation equation

Absorption rate = (HU value or BMD value at 1 week after surgery – HU value or BMD value at 3 and 6 months after surgery)/(HU value or BMD value at 1 week after surgery). The difference between each pair of absorption rates (DAR) was calculated as the absolute value of (BMD absorption rate at 3 and 6 months after surgery – HU value absorption rate at 3 and 6 months after surgery).

### Statistical analysis

Statistical Package for the Social Sciences version 26.0 (IBM Corp., Armonk, NY, USA) was used for statistical analysis. The intraclass correlation coefficient was used to verify the measurement stability of each study. Data were expressed as mean ± standard deviation. Correlation analysis was used to verify whether there was a correlation between the HU value and QCT BMD value and whether the HU value or BMD changed with time. The linear regression equation between HU and BMD was calculated using linear regression analysis. One-way analysis of variance was used to test whether there were significant differences in the HU or BMD values at different follow-up times. A paired-sample *t* test was used to verify whether the absorption rates of HU and BMD were consistent at the same follow-up. A correlation test was used to verify whether there was a correlation between sex, age, reconstruction site, tumor nature, body mass index (BMI), HU value, and BMD absorption rate. Statistical significance was set at *P* < 0.05.

## Results

A total of 54 patients (38 male and 16 female patients) were included in this study. The average age was 53.2 ± 14.4 years (range: 16–71 years). Preoperative fasting blood glucose was 5.6 ± 1.3 mmol/L (range 3.2–10.9 mmol/L). BMI was 23.8 ± 3.81 (range: 16.41–35.3). The average follow-up time was 5.1 ± 2.2 months (3–9 months) (Table 1). None of the patients underwent denture restoration postoperatively.

**Table 1:** Demographic information

Variable	
Number of patients (person)	54
Sex, *n*	
Male	38
Female	16
Age (years)	
Mean ± standard deviation	53.2 ± 14.4
Range	16–71
Age group, *n*	
0–19 years	3
20–39 years	8
40–59 years	21
> 60 years	22
Fasting blood glucose (mmol/L)	
Mean ± standard deviation	5.6 ± 1.3
Range	3.2–10.9
Blood glucose classification, *n*	
Normal blood glucose (< 6.1 mmol/L)	42
Hyperglycemia (> 6.1 mmol/L)	12
Reconstruction site, *n*	
Maxilla	8
Mandible	46
Tumor nature, *n*	
Benign	18
Malignant	36
BMI	
Mean ± standard deviation	23.8 ± 3.81
Range	16.41–35.3
BMI rating, *n*	
Normal weight (< 23.9)	29
Obesity (> 23.9)	22

*BMI* Body mass index

**Fig. 4:**
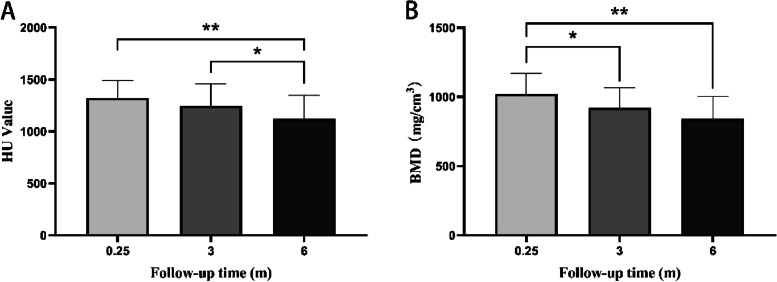
BMD and HU values at different follow-up times. **A** HU value. **B** BMD. BMD, bone mineral density; HU, Hounsfield unit. *P < 0.05, **P < 0.01

The fibular BMD was 1 020 ± 149.1 mg/cm^3^ at 1 week after the surgery, 920.9 ± 143.5 mg/cm^3^ at 3 months, and 842.1 ± 162.3 mg/cm^3^ at 6 months, and there was a significant difference between the value at 1 week and 3 months (*P* < 0.05) and the value at 1 week and 6 months (*P* < 0.01). HU value was 1 322.5 ± 165.9 at 1 week after the surgery, 1 246.4 ± 210.6 at 3 months, and 1 120 ± 228 at 6 months, there was a significant difference between the value at 1 week and 6 months (*P* < 0.01) and the value at 3 months and 6 months (*P* < 0.05) (Fig. 4).

**Fig. 5:**
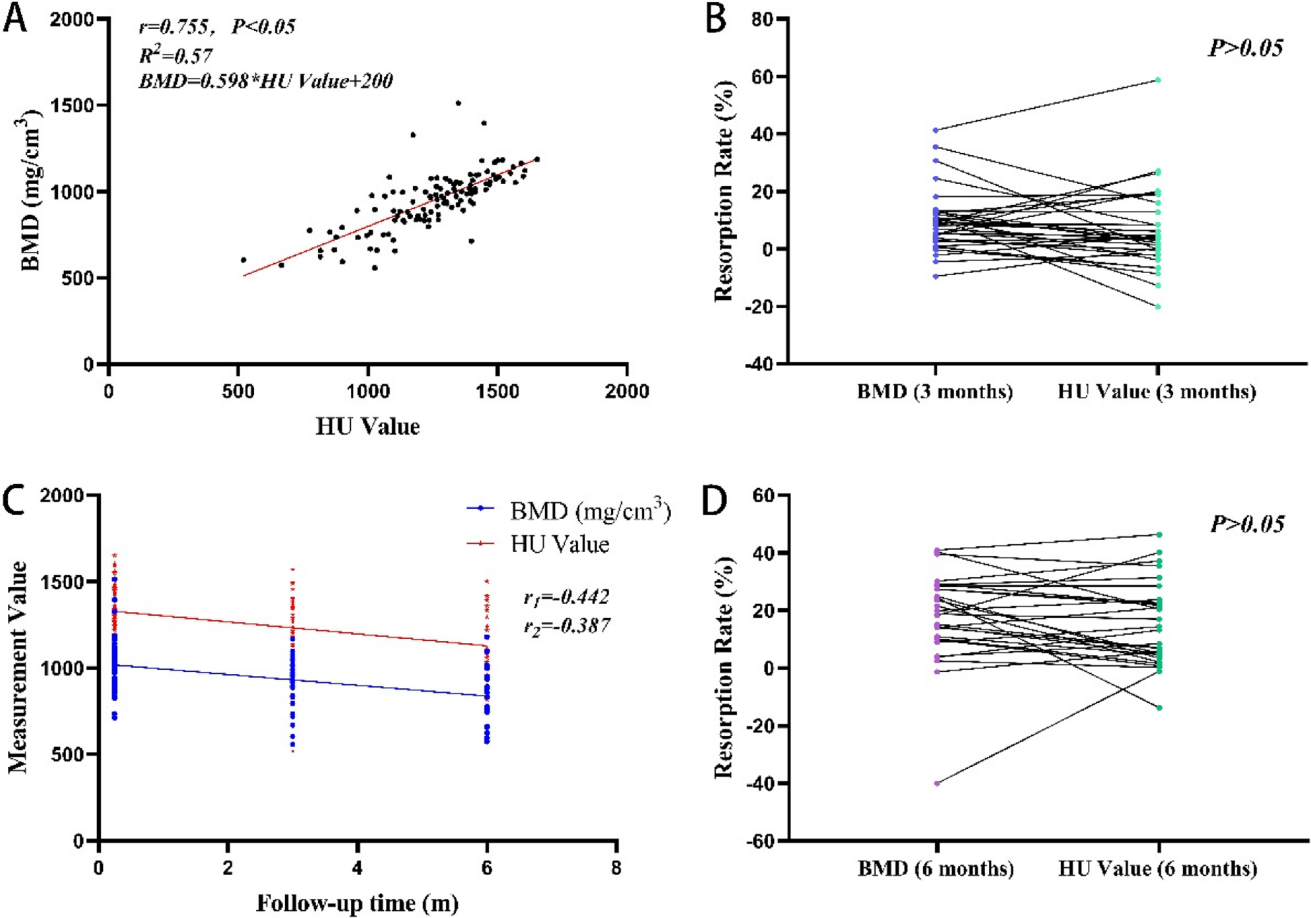
Comparison between HU value and BMD. **A** Correlation analysis and linear regression equation between HU value and BMD. **B** BMD and HU value absorption rate at 3 months after surgery. **C** HU value and the changing trend of BMD at different times. **D** BMD and HU value absorption rate at 6 months after surgery. BMD, bone mineral density; HU, Hounsfield unit

There was a significant correlation between the HU values and BMD (*P* < 0.01) (Fig. 5A). The linear regression model between the two values was statistically significant: BMD = 0.598 HU value + 200. The results showed no significant differences in absorption rates (Fig. 5B, D). We calculated the DAR and classified it into four levels: approximately 66.7% and 75.9% of patients had DAR < 10% at 3 and 6 months; however, approximately 12% and 13.8% of patients had DAR > 20% at 3 and 6 months, respectively (Table 2). The HU value and BMD significantly correlated with follow-up time (*P* < 0.01) (Fig. 5C).

**Table 2:** DAR classification

DAR (%)	At 3 months (%)	At 6 months (%)
< 10	66.7	75.9
10–20	21.2	10.3
20–30	6	6.9
> 30	6	6.9

The difference in absorption rate between BMD and HU values at 3 and 6 months was classified into four levels. The two columns on the right show the percentage of patients whose absorption rate difference value was within these levels (indicated in the first column) at 3 and 6 months, respectively

*DAR*, the difference between each pair of the absorption rate

According to the test results, sex was correlated with the absorption rate of BMD 6 months after surgery (*r* = 0.382, *P* < 0.05), and sex (*r* = 0.506, *P* < 0.05) and reconstruction site (*r* =  − 0.371, *P* < 0.01) were correlated with the absorption rate of the HU value 6 months after surgery. Other factors such as age, fasting blood glucose level, tumor nature, and BMI were not correlated with the absorption rate after surgery.

## Discussion

Miyamoto et al. found that the thickness of the fibular cortical bone is an important index for determining the initial stability of implants [8]. Therefore, previous studies on the measurement and evaluation of the quality of bone grafts after jaw reconstruction often focused on length, height, thickness, and other indicators of the fibula and iliac bone [9, 10]. BMD is another important indicator of the long-term survival rates of implants. Owing to the lack of recognized and reliable BMD measurement methods, few studies have evaluated BMD after jaw reconstruction. In recent years, with technological progress, this has become possible.

Currently, the mainstream methods for measuring BMD are dual-energy X-ray absorptiometry (DXA) and QCT [11]. DXA is widely used in clinical practice and is generally considered as the gold standard for the diagnosis of osteoporosis [12, 13]. QCT accurately measures BMD by calibrating the HU value in the CT image according to the calibration phantom, with a known density as the standard [14]. Compared with DXA, its measurement is closer to the physical definition of “density”, which is not affected by the shape, weight, and other factors. It can measure the BMD of the cortical and cancellous bones separately, which is the average density in a three-dimensional cylinder (mg/cm^3^) [11]. Löffler et al. confirmed that, compared with DXA measurement, QCT had more accurate results in measuring BMD and could also be used to predict possible spinal and vertebral fractures [15]. Owing to these advantages, QCT has become a reliable tool for the accurate measurement of jaw BMD. Maki et al. confirmed the feasibility of using QCT to measure jaw BMD [16].

HU value is also assumed to be likely to substitute BMD to some degree [17, 18]. Schreiber et al. conducted a comparative study on the correlation between the HU value and BMD of the vertebral body measured using DXA and concluded that the HU value could be used for opportunistic screening of osteoporosis but cannot replace DXA [7]. If the CT machine, environment, or scanning parameters change, HU values become inconsistent [19]. Studies have also shown that when measuring low-density substances, such as water and cancellous bone substitutes, the change rate of HU values between different CT machines is relatively low, which is within the acceptable range; however, for high-density substances such as the bone cortex, the measurement difference between different machines is more obvious [19].

Therefore, our study used the BMD obtained using QCT as the standard to explore whether HU values measured using the same CT machine could be used as an indicator to monitor changes in BMD in patients after jaw reconstruction. Our results showed that there was no significant difference between absorption rates (Fig. 5B, D); however, there were some specific points that showed apparent inconsistencies, and almost all pairs of points were not identical, indicating that HU values could not completely represent BMD. We also noticed that there was a significant difference in BMD but not in HU values between 1 week and 3 months, and there was a significant difference in HU values but not BMD between 3 and 6 months (Fig. 4), which could also imply that the two values were not completely equal. We also calculated the DAR and found that most of the absorption rate difference value was less than 10%; however, there was still some difference up to more than 30% (Table 2), reflecting that the HU value is close to the BMD obtained using QCT in the majority of situations; however, it may also vary sometimes.

The BMD values obtained in this study showed a downward trend over time. Some researchers have attempted to observe the morphological changes in the grafted fibula; however, they found that it changed marginally 1 year after surgery [20]. Kang et al. measured the thickness of the cortical bone of the fibula and found obvious absorption 1 year after surgery, with an average absorption rate of more than 20%, which is close to our results [4]. We also tried to measure the BMD of the cancellous bone in the center of the fibula. However, most of the results showed negative values, which are meaningless; therefore, we did not present these results in this study. There are many explanations for this decline in BMD. First, the lack of stress stimulation may be a primary factor. Wolff et al. confirmed that the bone will change according to the changes in the external environment [21, 22]. Owing to the lack of denture repair, the reconstruction site will not be stimulated by masticatory force, which will also lead to a decline in BMD. Muscle density, muscle size, and other factors may also affect BMD. After jaw reconstruction, patients may experience a large degree of change in weight and muscle mass within a short period [23, 24].

This study showed a significant correlation between HU values and BMD. Mohamed et al. measured HU values after fibula reconstruction and found that the HU value decreased at 6 months after surgery, with an average value of 961.23, which was slightly lower than the measurement results of this study [25], ascribed to the fact that different CT machines may obtain different HU values for their instability. Chen et al. attempted to use the HU value to evaluate the changing trend of the BMD of the iliac cancellous bone after jaw reconstruction and found that it can roughly reflect the bone resorption of the iliac cancellous bone [26]. Our study showed a similar decreasing trend of HU values and BMD over time; however, they were not completely consistent; therefore, we infer that HU values can roughly reflect the decreasing trend of fibula BMD. Studies have confirmed a linear relationship between HU value and BMD [27]. Therefore, we also calculated the linear regression equation between HU values and BMD, which was statistically significant.

The limitations of this study are that due to the complex situation of patients after jaw reconstruction, metal products such as titanium plates and titanium nails were avoided in the measurement process; however, the impact of these artifacts on the measurement results is not yet known, and further research is needed to confirm this in the future.

## Conclusions

Therefore, this study draws the following preliminary conclusions. There is a significant correlation between HU values and BMD. In clinical practice, patients often require CT for routine examination. Thus, the HU value can be used to evaluate fibula BMD and reflect its changing trend roughly in a group of patients as opposed to just a single person because it may sometimes produce some apparent error.

## Data Availability

The data is available from the corresponding author upon reasonable request.

## Abbreviations

BMD: Bone mineral density
HU: Hounsfield unit
QCT: Quantitative computed tomography
ROI: Region of interest
DAR: Difference between each pair of absorption rates
BMI: Body mass index
DXA: Dual-energy X-ray absorptiometry
CT: Computed tomography
PACS: Picture Archiving and Communication System
DICOM: Digital Imaging and Communications in Medicine
